# Expression Regulation and Function of T-Bet in NK Cells

**DOI:** 10.3389/fimmu.2021.761920

**Published:** 2021-10-05

**Authors:** Chen Huang, Jiacheng Bi

**Affiliations:** CAS Key Laboratory of Quantitative Engineering Biology, Shenzhen Institute of Synthetic Biology, Shenzhen Institute of Advanced Technology, Chinese Academy of Sciences, Shenzhen, China

**Keywords:** Tbx21, Eomes, Th1, tumor immunology, immunotherapy

## Abstract

Natural killer (NK) cells are cytotoxic innate lymphocytes that play an important role in immune surveillance. The development, maturation and effector functions of NK cells are orchestrated by the T-box transcription factor T-bet, whose expression is induced by cytokines such as IFN-γ, IL-12, IL-15 and IL-21 through the respective cytokine receptors and downstream JAK/STATs or PI3K-AKT-mTORC1 signaling pathways. In this review, we aim to discuss the expression and regulation of T-bet in NK cells, the role of T-bet in mouse NK cell development, maturation, and function, as well as the role of T-bet in acute, chronic infection, inflammation, autoimmune diseases and tumors.

## Introduction

The transcription factor T-bet (also known as *Tbx21*) is an important transcription factor for the immune system, orchestrating multiple types and aspects of immune responses. T-bet belongs to the T-box subfamily, Tbr1, which possesses a 180-200-amino-acid-residue conserved sequence coding for a T box domain for binding of the DNA consensus sequence AATTTCACACCTAGGTGTGAAATT (1). T box domain of T box proteins might bind the target sequence as a dimer, interacting with both the major and the minor grooves of the DNA (2). In addition to the DNA –binding domain (T-box), T box proteins have transcription activation or inhibition domains (3, 4). T-bet is mainly expressed in the lung, thymus, and spleen as determined by Northern blot analysis of multiple organs (5). T-bet is not expressed in resting splenocytes, but is specifically expressed in immune cells such as Th1, NK, B and DC cells after stimulation (5, 6). *Tbx21*, the gene encoding T-bet, was discovered in an attempt to isolate transcription factors that can direct the tissue-specific expression of Th1 cytokines. The transcription factor was therefore named *T-bet*, for T box expressed in T cells (5, 7). Later, T-bet expression and function were further revealed in other lymphocyte populations (8).

NK cells are cytotoxic innate lymphocytes, which mainly develop and start the process of maturation from the bone marrow (9), and play an important role in the early defense of intracellular pathogens and immune surveillance of tumors, by rapid production of IFN-γ, as well as other important cytokines and chemokines, and by cytotoxicity against infected or transformed cells (10, 11). Unlike T cells, NK cells do not require priming before displaying cytolytic activity against target cells. Due to the anti-tumor potential of NK cells, NK-based tumor immunotherapy has been an important arm of current immunotherapy research. However, improved efficacy of NK-based immunotherapy requires a better understanding of NK cell biology.

T-bet is a central transcription factor for NK cells, governing multiple processes including the development, maturation and function of NK cells. The expression of T-bet is dynamically and finely regulated in various disease conditions, as well as in different development stages or functional status of NK cells, which should impact its role in regulating NK cell activity. In this review, we’ll discuss the expression regulation and functions of T-bet, with an emphasis on NK cells. Based on the knowledge of the expression regulation and function of T-bet in NK cells, we also discuss potential immunotherapy strategies that could exploit the anti-tumor capacity of NK cells in future.

## An Overview of T-Bet in the Immune System

### T-Bet in NK Cells

T-bet is a key transcription factor for natural killer cell maturation (12). T-bet expression correlates with NK cell maturation during the process from less mature CD11b^pos^CD27^pos^ stage to the more mature CD11b^pos^CD27^neg^ stage. Deficiency of T-bet leads to significantly reduced NK cell numbers, as well as the lack of TRAIL^+^DX5^-^ NK cells. In addition, Eomes–deficient NK precursors could not develop into NK cells if T-bet is also deficient, as NK cells could not be detected in any organs in *Tbx21^−/−^Eomes^Flox/Flox^; Vav-Cre^+^* mice (13). Therefore, T-bet coordinates with Eomes for the development of NK cells, and T-bet is required for NK cell maturation (14–16). T-bet is also important for NK cell effector functions. Although T-bet^-/-^ NK cells can also rapidly secrete IFN-γ compared with wild-type NK cells, the maintenance of IFN-γ production is impaired in the absence of T-bet. Besides, the cytolytic activity of T-bet^−/−^ NK cells against target cells was significantly reduced (14). The above aspects will be discussed in detail in the later sections of this review.

### T-Bet in T Cells

T cell differentiation is essential for the outcome of immune responses in diseases (17). T-bet plays an important role in the regulation of differentiation of T cell subsets, which is orchestrated by the activity of a series of transcription factors (18). T-bet promotes Th1 differentiation, but inhibits Th2, Th17 and Tfh cell lineage commitment (6, 19). T-bet regulates Treg cell homeostasis and promoted T-bet^+^ Treg cells to accumulate at sites of Th1-mediated inflammation by inducing expression of the chemokine receptor CXCR3 on Treg cells (20). CD8^+^T cells that have high T-bet levels tend to become terminally differentiated effector cells, whereas the cells that have low T-bet levels have a higher memory cell developmental potential (21).

### T-Bet in Other Immune Cells

In addition to NK cells and T cells, T-bet also regulates other lymphocyte populations. T-bet acts as a selective inducer for the expression of *Iγ2a* transcription, and promotes IFN-γ-mediated IgG2a class-switching in B cells (22). IFN-γ/T-bet–dependent pathway regulates CXCR3 expression in B cells to drive the migration of memory B cells to inflammatory foci (23). On the other hand, T-bet is expressed in DCs at levels comparable with Th1 cells and is necessary for the production of IFN-γ and activation of antigen-specific Th1 cells (24). Moreover, T-bet suppresses intestine IL-17A –producing ILCs, which are potent promoters of ulcerative colitis, by decreasing the expression of IL-7 receptor in these cells (25). IL-2 and IL-15 are structurally related cytokines that share common receptor subunit-CD122 (IL-2 receptor β chain) (26). T-bet is required for optimal CD122 expression on thymic intraepithelial lymphocyte precursors (12), as well as on Vα14i NKT cells (14) for their development or maturation.

## Regulation of T-Bet Expression

### T-Bet Expression Is Induced by IFN-γR/STAT1 Pathway

IFN-γ plays a critical role in regulation of Th1 cell development (27). After 6 or 24h of anti-CD3 stimulation, IFN-γ -producing Th1 cells, but not Th2 cells, increased T-bet expression. After infection of a prototypical Th1-inducing pathogen, *Toxoplasma gondii*, splenic *Tbx21* mRNA levels increased 4-fold in wild type mice, but not in *Ifnγ* ^−/−^mice, indicating that IFN-γ is essential in inducing T-bet expression (28). Naive CD4^+^ T cells from lymph nodes of 5C.C7 T cell receptor transgenic/*Rag2*-deficient mice incubated with APCs increased expression of T-bet in T cells in the presence, but not in the absence, of TCR –specific peptide and IFN-γ, indicating that T-bet could be induced in response to TCR stimulation in an IFN-γ–dependent manner (28). Furthermore, IFN-γ stimulation failed to induce T-bet in *Stat1*-deficient mice (29). The above evidence suggests that T-bet is induced in T cells by TCR through the IFN-γR/STAT1 signaling pathway.

Moreover, three potential T-box binding sites were detected in *Ifnγ* gene, two of which were located in the proximal promoter region and one in the third intron (5), showing that T-bet is a transactivator of the *Ifnγ* gene and mediates positive feed-forward regulation of endogenous IFN-γ production.

### T-Bet Expression Is Induced by IL-12R/STAT4 Pathway

**Figure 1:**
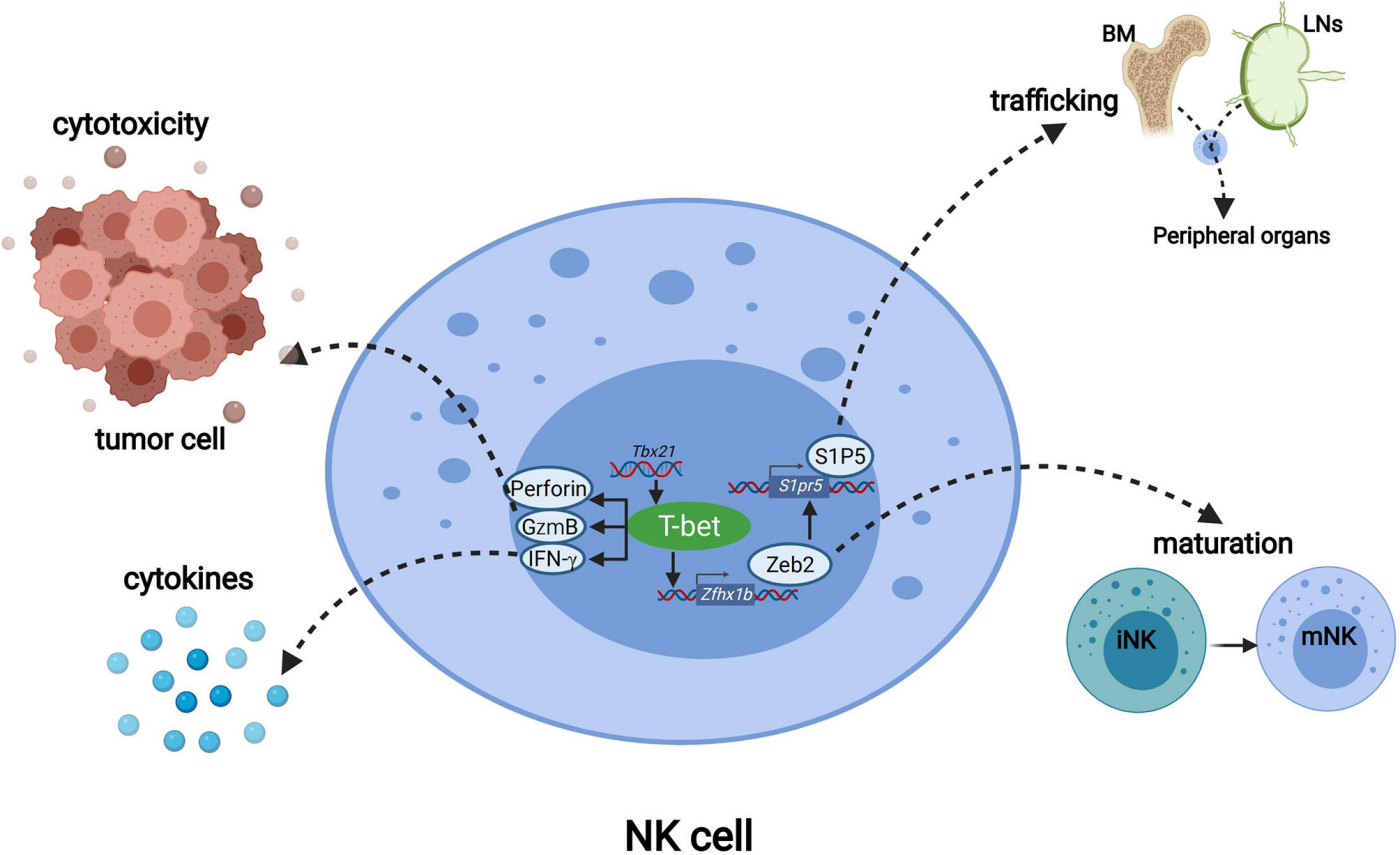
The regulation of T-bet expression in NK cells. IFN-γ binds to IFN-γR and regulates the expression of T-bet through JAK-STAT1 pathway. IL-12 induces T-bet expression independent of IFN-γ through JAK-STAT4 pathway. IL15 activates PI3K-AKT-mTORC1signaling axis to promote the expression of T-bet, whereas Foxo1 downstream of mTORC2 can inhibit the expression of T-bet in NK cells. IL-21 can activate the JAK-STAT3 pathway, and the activated STAT3 combines with MyD88 to activate the downstream NK-κB pathway to up-regulate the expression of T-bet.

Besides IFN-γ, IL-12 is also involved in the differentiation of naive T lymphocytes into Th1 cells (30, 31), and could also induce the expression of T-bet. Although IL-12 could be an inducer of IFN-γ, *Ifngr* ^−/−^ T cells increased T-bet expression after stimulation with IL-12 (32), indicating that IL-12 induces T-bet expression independent of IFN-γ. In support of this, chromatin immunoprecipitation showed that the conserved T-bet enhancer element 13 kilobases upstream of the transcriptional start site can interact with IL-12–activatedSTAT4 to induce T-bet expression (33). Purified splenic NK cells from wild-type, *Ifnγ^−/−^*, and *Stat4^−/−^* mice stimulated with IL-12/IL-18 for 12h showed that IL-12 induced the expression of T-bet through STAT4 independently of IFN-γ (14). Therefore, early after TCR stimulation, T-bet expression is mainly induced through the TCR/IFN-γ signaling pathway, when the expression of IL-12 receptor β2 subunit is repressed by TCR signaling, whereas in the later phase, this repression is removed upon termination of TCR stimulation, allowing T-bet induction through STAT4 downstream of IL-12R signaling pathway (**Figure 1**).

### T-Bet Expression Is Induced by TLR/MyD88 Pathway

MyD88 is a TIR-domain-containing adaptor protein in response to Toll-like receptors in TLR signaling pathway (34). MyD88 is located downstream of the TLR (except TLR3) and IL-1 receptor family, and transmits signals to activate NF-κB and MAP kinases and to induce inflammatory cytokines (35). 3 weeks after naïve CD4^+^ T cells from WT donors were transferred into congenic WT or MyD88 deficient recipient mice, the differentiation of T-bet^high^ memory-phenotype (MP) cells was significantly fewer in MyD88 deficient recipient mice than in WT mice, which indicates that extrinsic TLR-MyD88 signaling plays a key role in driving MP cells to differentiate into T-bet^high^ subpopulations (36). B cell TLR signaling has been reported to induce T-bet expression to produce IgG2A/cAb in response to viral infection (37, 38). Immunization with Qβ-VLP of irradiated chimeric mice of mixed bone marrow cells from WT (CD45.1^+^) and MyD88 KO (CD45.2^+^) showed that T-bet expression in MyD88 KO derived Qβ^+^ B cells from chimeric mice was much lower than that in WT-derived cells, which suggests that B cell-intrinsic MyD88 signaling was required for T-bet expression (39). On the other hand, IL-21 can induce STAT3 to bind to MyD88 activated sequence (GAS) and SIE (a well-characterized element binding activated STAT3), leading to up-regulation of T-bet expression in NK cells (40). This indicates that IL-21 may regulate T-bet expression in NK cells through the JAK-STAT3-MyD88 axis.

### T-Bet Expression Is Promoted by mTOR Signaling Pathway

**Figure 2:**
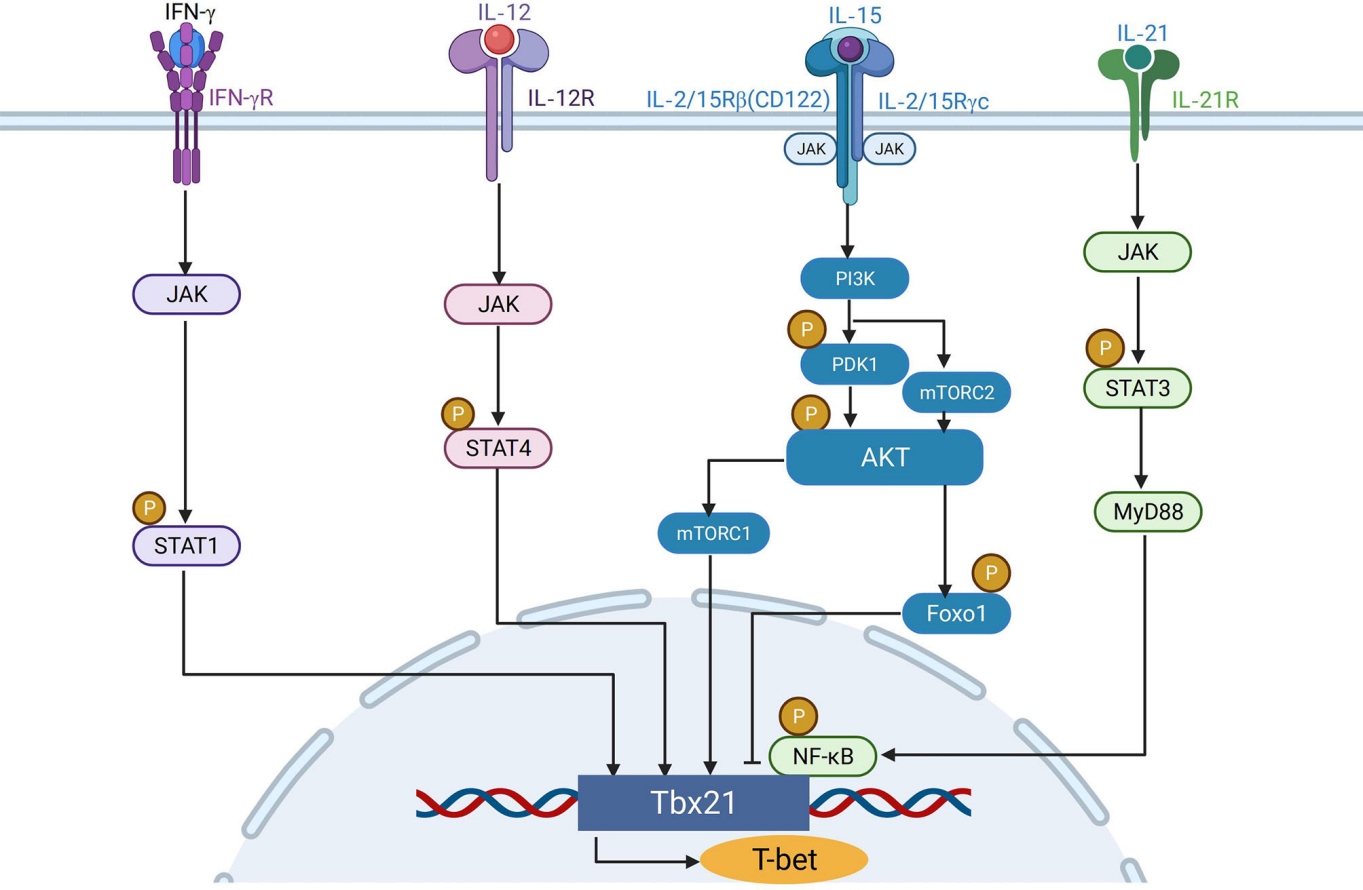
The functions of T-bet in NK cells. T-bet promotes the transcription of genes including *Prf1*, *Gzmb* and *Runx1* to mediate NK cell cytotoxicity. T-bet positively regulates the secretion of IFN-γ to regulate immune responses. Through inducing the expression of Zeb2, T-bet promoted the differentiation from immature NK cells to mature NK cells. T-bet induces Zeb2, and Zeb2 binds to the *S1pr5* promoter which promotes mature NK cells egress from lymph nodes and bone marrow to peripheral organs.

mTOR binds to Raptor and Rictor to form two complexes, mTORC1 and mTORC2, respectively (41). The activity of mTOR could be stimulated *via* IL-15 signaling in NK cells (42), which plays an important role in NK cell effector functions (43). The absence of either mTORC1 or mTORC2 led to decreased expression of T-bet by NK cells (44, 45). Tsc1 (a repressor of mTOR) negatively regulated IL-15-triggered mTORC1 activation in NK cells. TSC1 deficiency resulted in higher activity of mTORC1 in *Tsc1* ^−/−^ NK cells than in WT NK cells. Meanwhile, T-bet was expressed at a higher level in *Tsc1* ^−/−^ NK cells compared with WT NK cells (46). Six phosphorylation sites of T-bet were identified to be targets of mTORC1, among which at least three sites were required for recruitment of chromatin remodeling complexes to the *Ifng* gene promoter by T-bet for a normal level of IFN-γ production (47). These studies indicate that T-bet expression is promoted by mTOR signaling pathway (**Figure 2**).

### Posttranslational Protein Modification of T-Bet

The applications of T-bet reporter mice have facilitated the determination of T-bet expression level (6), which, together with intracellular staining for flow cytometry, have enabled the detection of T-bet expression level even in small lymphocyte populations that are difficult for western blot or Q-PCR. However, the activity of T-bet is regulated by posttranslational protein modifications (48), which could have made it more complex to assess T-bet activity. For example, IL-2 –inducible tyrosine kinase (ITK) phosphorylates the tyrosine residue Y525 of T-bet in Th cells after stimulation with TCR and IL-12, which is important for interaction with GATA-3 and for suppression of Th2 cell differentiation (49). In addition to phosphorylation by ITK, tyrosine residues Y219, Y265 and Y304 of T-bet are also phosphorylated by c-Abl, which is essential for IFN-γ production and suppression of Th2 cytokine production, and for control of allergic lung inflammation (50). Moreover, serine residue S508 phosphorylation is important for T-bet to interact with and prevent NF-κB p65 from binding to the *Il2* promoter and for T-bet mediated suppression of IL-2 production in T cells (51). While the phosphorylation of tyrosine or serine residues in T-bet regulates the activity of T-bet, ubiquitination of lysine residues in T-bet affects both the activity and half-life of T-bet. Among 16 lysine residues of T-bet, although ubiquitination of the lysine residue K313 leads to proteasomal degradation of T-bet, such modification is important for T-bet’s DNA binding activity and T-bet –mediated suppression of IL-2 and Th2 cytokines (52). Future studies are required to determine the impact of posttranslational protein modifications of T-bet on NK cell biology.

## The Role of T-Bet in NK Cell Immunity

### T-Bet Maintains the Stability of Tissue-Resident NK Cells

T-bet was proposed to maintain the stability of immature NK cells, since TRAIL^+^DX5^-^ NK cells were dramatically reduced in *Tbx21−/−* mice. Deletion of T-bet by treating *Tbx21 ^flox/flox^* NK cells with TAT-Cre ex vivo resulted in the reduction of this population after transferred into *Il2rg^−/−^Rag2^−/−^* mice, confirming that T-bet stabilizes this NK cell subset (13). However, lack of T-bet was later shown to significantly reduce TRAIL^+^CD49a^+^CD49b^-^ tissue-resident NK cells (trNK cells), which, though showing an immature phenotype, represents a lineage distinct from circulating conventional CD49a^-^CD49b^+^TRAIL^-^ NK cells (53, 54). Therefore, the developmental stability of trNK cells is dependent on T-bet.

### T-Bet Is Required for NK Cell Maturation

scRNA-seq revealed that more than 65% of T-bet-deficient NK cells were classified as the least mature iNK cluster, and the expression of immature NK signature genes was highly upregulated in T-bet-deficient NK cells (55). This shows that in the absence of T-bet the overall NK cell population displayed a more immature phenotype, indicating that T-bet is required for NK cell maturation. Mechanistically, T-bet induces and cooperates with Zeb2 to promote the transcription program for NK cell maturation, and induces S1P5 for mature NK cell egress from the bone marrow.

#### T-Bet Induces and Cooperates With Zeb2 to Promote NK Cell Maturation

The transcription factor Zeb2 (zinc finger E-box–binding homeobox 2) was among the most highly induced transcription factor during NK cell maturation. The mRNA expression level of *Zeb2* in more mature CD27^-^NK cells is significantly higher than that in less mature CD11b^-^and DP NK cells. The number of mature CD27^-^NK cells in the spleen and bone marrow of NK-*Zeb2* ^Tg/+^ and NK-*Zeb2* ^Tg/Tg^ mice is higher than that in wild-type mice, while the number of immature CD11b^-^ and DP NK cells in these mice is lower. Therefore, Zeb2 expression promotes the maturation of NK cells. From percentage and absolute numbers of NK cells in the spleen and their maturation status as assessed by CD11b/CD27 staining, the phenotypes of mature NK cells from *Tbx21* ^-/-^ and *Zeb2* ^-/-^mice were similar. The NK cells from *Tbx21* ^-/-^, *Tbx21* ^+/-^, WT and *Tbx21* transgenic mice showed graded expression levels of *Zeb2* mRNA in NK cells correlating with graded doses of T-bet (56). These findings indicate that Zeb2 acted downstream of T-bet, and that T-bet is necessary to induce Zeb2 expression. Hence, T-bet promotes terminal NK cell maturation by cooperating with Zeb2 (**Figure 2**).

#### T-Bet–Dependent S1P5 Promotes Egress of Mature NK Cells

Sphingosine-1-phosphate receptor 5 (S1P5), encoded by the gene *S1pr5*, is required for mature NK cell egress from the bone marrow to the peripheral (**Figure 2**). Chimeric mice with S1P5 deficient and wild-type bone marrow cells demonstrated a significant accumulation of S1P5-deficient NK cells in the BM and the lymph nodes (LNs). The ratio of S1P5deficient NK cells to wild-type NK cells in the lymphocytes was lower in the peripheral than in the LN (57–59). The expression level of *S1pr5*mRNA in T-bet deficient NK cells was significantly lower than that in wild-type NK cells (57). The expression of *S1pr5* is induced by the positive feedback loop of T-bet and Zeb2, in which T-bet induces Zeb2, and Zeb2 binds to the *S1pr5* promoter to initiate transcription (56, 60).

### T-Bet Is Required for NK Cell Effector Functions

#### T-Bet Is Required for Sustained IFN-γ Production by NK Cells

IFN-γ is a critical effector cytokine produced by NK cells, playing important role in anti-tumor and anti-viral immune responses (61). In the absence of T-bet, early IFN-γ production by NK cells is not affected. However, after 24 hours of stimulation, the level of IFN-γ produced by T-bet^-/-^NK cells was significantly lower than that of WT mice. These findings indicate that the early and rapid secretion of IFN-γ is independent of T-bet, but T-bet expression is required for the maintenance of IFN-γ production by NK cells (14).

#### T-Bet Is Required for NK Cell Cytolytic Activity

In addition to the production of IFN-γ, the major effector function of NK cells also includes rapid production of perforin and granzymes for cytolysis of virally-infected or malignant cells (62–64). ChIP analysis of IL-12/IL-18-activated murine LAK cells showed that T-bet could bind to the promoter of *Gzmb*, *Prf1* and *Runx1*. Both in resting NK cells and in IL-12 & IL-18 –stimulated NK cells, deficiency of T-bet led to decrease mRNA levels of these three genes (14). Therefore, T-bet promotes the transcription of genes including *Prf1*, *Gzmb* and *Runx1*to mediate NK cell cytolytic activity (**Figure 2**).

## The Role of T-Bet in Diseases

### T-Bet in Acute Infections

T-bet plays an important role in immune response against acute infections. In the process of infection with the intracellular bacterial pathogen Listeria monocytogenes (LM), T-bet enhances its own expression and IFN-γ production through the STAT1 signaling pathway, while the absence of T-bet may impair sustained secretion of IFN-γ by NK cells and T cells (65–67). In acute infection with LCMV Armstrong, loss of T-bet expression in Tfh cells suppressed Tfh expansion, and reduced the levels of germinal center B cells and plasma cells, as well as lower LCMV-specific IgG and subtype IgG2c titers in serum, indicating that T-bet is essential for Tfh response and antibody IgG2 class switching in acute LCMV infection (68). In addition, T-bet^-/-^mice displayed a compromised resistance to Mycobacterium tuberculosis infection, mainly due to impaired IFN-γ production from the CD4^+^Th1 T cell subset to form an effective type 1 immune response (69, 70). During the infection of intracellular parasite Toxoplasma gondii, dendritic cells and macrophages produce IL-12 to promote the activation and expansion of NK cells and T cells populations expressing high levels of T-bet and IFN-γ (71–75).

### T-Bet in Inflammatory and Autoimmune Diseases

T-bet is critical for the development of immunopathology in the experimental autoimmune encephalomyelitis (EAE) model of inflammatory demyelination (76, 77). Importantly, T-bet–dependent NKp46^+^ ILCs are required for the initiation of CD4^+^ Th17 –mediated neuroinflammation (78). In Crohn’s disease, a chronic inflammatory disease of the gastrointestinal tract, T-bet protein expressed higher in lamina propria CD4^+^ T cells from patients than from healthy controls. The high expression of T-bet accelerates the development of Th1-mediated colitis, whereas mice lacking T-bet are more likely to develop Th2-mediated colitis (79, 80). In heart failure(HF), TCR –activated PKC-θ induces T-bet, which ultimately leads to the stimulation of immune response and the occurrence of cardiac hypertrophy and fibrosis (81). However, T-bet could act as a double-edged sword in response to inflammatory and autoimmune diseases as well. In cardiovascular disease (CVD), on the one hand, T-bet stimulates and activates the functions of immune cells such as NK and DC to increase inflammation and cardiac injury. On the other hand, T-bet activates regulatory T cells and stimulates angiogenesis to inhibit inflammation and replaces damaged vessels with new vessels (82). Besides, T-bet in B cells can regulate immunoglobulin (IgG) class switching. Increased T-bet expression in B cells inhibits IgE production and alleviates IgE-mediated allergic reactions and asthma (22, 83).

### T-Bet in Tumors and Chronic Infections

T-bet-deficient mice generated in a transgenic adenocarcinoma mouse prostate model (TRAMP) indicated that lack of T-bet has little effect on the development of primary tumors, but that T-bet is needed to suppress metastasis (84). In addition, T-bet-deficient mice were extremely sensitive to the metastasis and spread of B16F10 melanoma cells (85). On the other hand, T-bet was down-regulated in adoptively transferred NK cells upon exposure to tumors and proliferation, which was associated with down-regulation of activating receptors and IFN-γ (86). T-bet mRNA level expressed by the exhausted NK cells of HBV individuals is lower than that of the NK cells of healthy donors (87). T-bet expression in virus-specific CD8^+^ T cells is also reduced during chronic infections, which negatively correlated with the expression of exhaustion marker PD-1 (88). Therefore, T-bet is critical for the control of tumors and chronic infections, but its expression might be downregulated in effector cells in these contexts.

## Perspectives

### T-Bet in NK–Based Immunotherapy

In NK-based tumor immunotherapy, the following strategies are currently under investigations to promote the anti-tumor effects of NK cells: 1.Blockade of the cell surface or intracellular inhibitory checkpoint molecules of NK cells (89–92); 2. Arming with chimeric antigen receptor (CAR) to improve tumor recognition specificity of NK cells (93, 94); 3. Bispecific antibody to connect NK cell activating receptor to target cells (95). Based on these technologies, as well as the knowledge on T-bet regulation and functions in NK cells, we can design strategies to promote the anti-tumor efficacy of NK –based immunotherapy.

Previous studies have shown that adoptive NK cell immunotherapy failed to effectively control tumors, which was associated with down-regulation of T-bet. Accordingly, we can further dissect the factors (inhibitory receptor-ligand interactions/soluble factors) responsible for the down-regulation of T-bet in tumor-associated NK cells, and design strategies to recover T-bet expression for maintaining the anti-tumor functions in NK cells. In line with this, small molecules potentially enhancing T-bet expression have been reported (96). Alternatively, ectopic expression of specific transcription factors in immune cells for tumor immunotherapy has also been reported (97, 98), T-bet –overexpressing CAR-T cells exhibited a Th1 phenotype with more effective anti-tumor activity, leading to improved survival of tumor-bearing mice (99). These studies suggest that the ectopic expression of T-bet in NK cells could be tested for tumor immunotherapy in future. In addition, based on knowledge on the cytokine receptors and signaling pathways responsible for inducing T-bet, we can rationally design the signaling domain of CAR-NK to trigger the T-bet –inducing pathways to promote the expression of T-bet for enhancing NK cell effector functions. Alternatively, we can design multi-specific antibody to trigger T-bet –inducing cytokine receptors to promote the expression of T-bet.

### Open Questions/Future Studies on T-Bet in NK Cell Biology

T-bet and Eomes are highly homologous transcription factors, which play redundant or non-redundant roles as key checkpoints in the regulation of NK maturation (13). The synergistic cooperation between the two can better exert the effector function of NK cells for the control of tumors and infections. The balance between T-bet and Eomes in NK cell maturation, differentiation and effector function still requires further in-depth studies for the spatiotemporal effects of these transcription factors, the transcription elements that regulate the transcription of T-bet and Eomes, and the molecular chaperones that help them fold and assemble at the protein level. On the other hand, the regulation of T-bet expression in NK cells is closely related to the pathogenesis of various diseases. Further studies on the expression of T-bet in NK cells in disease models will better demonstrate the role of T-bet in NK cell biology. T-bet downregulation was shown to be one of the hallmarks of NK cell exhaustion in tumors, whose details still lack further elaboration, and might benefit future design of NK-based immunotherapy. Moreover, the human TBX21 gene has 40 known polymorphisms, of which one has been shown to be related to the susceptibility to type 1 diabetes (100), and the other is related to the incidence of herpes simplex virus 2 (101). However, the effect of T-bet polymorphisms on NK–dependent immune responses in human diseases is largely unknown. Therefore, from the angle of T-bet, our knowledge on NK cell biology is still limited. Further research on T-bet is essential, not only to expand our understanding of NK cells, but also to assist the design of future strategies of immunotherapy.

## Author Contributions

CH and JB conceived and wrote the manuscript. All authors contributed to the article and approved the submitted version.

## Funding

This work was supported by National Key R&D Program of China 2020YFA0710802 (JB), Natural Science Foundation of China 82071768 (JB), and Natural Science Foundation of Guangdong Province 2019A1515011412 (JB).

## Conflict of Interest

The authors declare that the research was conducted in the absence of any commercial or financial relationships that could be construed as a potential conflict of interest.

## Publisher’s Note

All claims expressed in this article are solely those of the authors and do not necessarily represent those of their affiliated organizations, or those of the publisher, the editors and the reviewers. Any product that may be evaluated in this article, or claim that may be made by its manufacturer, is not guaranteed or endorsed by the publisher.
